# Comparison of Interleukin-6 with Other Markers in Diagnosis of Ovarian Cancer

**DOI:** 10.3390/jpm13060980

**Published:** 2023-06-11

**Authors:** Magdalena Bizoń, Zofia Awiżeń-Panufnik, Włodzimierz Sawicki

**Affiliations:** 1 Chair and Department of Obstetrics, Gynecology and Gynecological Oncology, Medical University of Warsaw, 03-242 Warszawa, Poland; 2 LUX MED Oncology Hospital, sw. Wincentego 103, 03-291 Warszawa, Poland

**Keywords:** ovarian cancer, diagnosis, interleukin-6, ovarian tumor markers

## Abstract

**Simple Summary:**

Ovarian cancer is difficult to diagnose at an early stage. The lack of symptoms leads to late diagnosis and a poor prognosis. Inflammation seems to be a basic mechanism of this disease. Searching for novel markers in ovarian cancer is important for clinical application. Nowadays, the most common markers are CA125 and HE4, but novel ones are still sought. According to the theory of the inflammatory background of neoplastic diseases, inflammatory markers such as C-reactive protein, procalcitonin and interleukin-6 have a potential prognostic role in diagnosis of ovarian cancer.

**Abstract:**

The lack of specific symptoms in ovarian cancer delays onset of the diagnostic process. Hence, most cases are recognized in late stages of the disease. The aim of this study was to confirm the role of Il-6 compared to other markers in diagnosis and survival in ovarian cancer. The database was collected from 13 January 2021 to 15 February 2023. In total, 101 patients with pelvic tumors with a mean age of 57.86 ± 16.39 participated in the study. In every case, CA125, HE4, CEA, CA19-9, Il-6, C-reactive protein and procalcitonin measurements were taken. Patients with ovarian borderline tumor and metastatic ovarian tumors were excluded from further analysis. Statistically significant correlations were found between diagnosis of ovarian cancer and levels of CA125, HE4, CRP, PCT and Il-6. Comparison of Il-6 with other markers revealed that longer overall survival correlated with lower values of Il-6. In the case of a higher concentration of Il-6, OS and PFS were shorter. Sensitivity and specificity of Il-6 in diagnosis of ovarian cancer were 46.8% and 77.8%, respectively, while for CA125, CRP and PCT were 76.6% and 63%; 68% and 57.5%; 36% and 77%, respectively. More investigations are needed to identify the most specific and sensitive marker for ovarian cancer.

## 1. Introduction

Ovarian cancer is one of the most common gynecological cancers in Poland. Unfortunately, a high rate of mortality is still observed [1]. According to worldwide data, morbidity and mortality have not decreased [2,3]. Ovarian cancer is the fifth cause of death among all malignant tumors [4].

The lack of specific symptoms in ovarian cancer delays the onset of the diagnostic process. This is why most cases are detected in late stages of the disease. Diagnosis in the early stage of the disease allows for longer overall survival and progression-free survival.

Unfortunately, there is no specific marker for diagnosis of ovarian cancer. One of the most common markers is CA125. It is not a specific marker, because elevated values are also observed in pregnancy, endometriosis, myomas and liver failure. Only in every second case in the early stage is it increasing [5]. In advanced stages of ovarian cancer, the level of CA125 is elevated beyond the normal range in about 80% of cases [6]. For better detection of ovarian cancer, the ROMA calculator was created to assess the risk of ovarian cancer based on the values of CA125 and HE4. Its sensitivity is estimated as 84% and specificity as 84% [7]. Ain et al. revealed the role of urine and a panel of serum biomarkers in detection of the early stage of ovarian cancer, but it is still insufficient [8].

Nowadays, new markers are still being sought. Difficulties in this area follow from the unknown pathophysiology of ovarian cancer. There is a hypothesis about incessant ovulation which causes damage and wound repair of the ovarian epithelium and an increased risk of oncogenesis. Oncogenesis in the ovarian epithelium is induced by inflammation. On the other hand, every situation of less ovulation can decrease the risk of ovarian cancer, such as pregnancy, oral contraceptive use or tubal ligation [9,10,11].

In light of the theory of an inflammatory mechanism in ovarian cancer, proinflammatory markers should be examined.

The role of interleukin-6 was for the first time demonstrated in 1990 by Van Meir in glioblastoma. Then, in 1992, Reibnegger identified it as a marker of progression in multiple myeloma. In the same year, Blay et al. revealed Il-6 as a prognostic marker in renal cancer [12,13,14].

Interleukin 6 (Il-6) is a pleiotropic cytokine taking part in inflammatory stimulation. The first clone and report of the human gene Il-6 was in 1986. It is located on chromosome 7p15-21 and consists of five exons and four introns [15]. Il-6 is produced by lymphocyte and non-lymphocyte cells and activates the innate immune system during infection or trauma [16,17,18].

Il-6 via the membrane receptor activates non-receptor tyrosine kinases including JAK2, which induce the JAK2/STAT3 cascade for angiogenesis and tumor enlargement because of regulating progression of the cell cycle. This mechanism is especially significant in cachexia, migration of tumor cells and progression of cancer [19,20]. Chronic inflammation is observed in a quarter of cancers [21]. The mechanism of Il-6 is highly activated in various neoplastic diseases such as breast cancer, pancreatic cancer, digestive cancers (gastric, colon, colorectal), lung and ovarian cancer [22,23,24].

The aim of this study was to confirm the role of Il-6 in the diagnosis of ovarian cancer and its prognostic role in overall survival.

## 2. Materials and Methods

### 2.1. Patients’ Characteristics

The single-center study was conducted in the Clinic of Obstetrics, Gynecology and Gynecological Oncology of the Medical University of Warsaw. The database was collected from 13 January 2021 to 15 February 2023. In total, 101 patients with pelvic tumors with a mean age of 57.86 ± 16.39 (range, 23.97–92.53 years) participated in the study. Median age was 60.17 years. In every case, transvaginal ultrasound was performed to confirm the presence of a pelvic tumor according to IOTA algorithms (GE Voluson S8 BT16). 

Inclusion criteria were: age above 18 years old, presence of pelvic tumor, no history of cancers in the past, no use of NSAIDs for 4 weeks before participating in the study, no inflammation diseases and no infection within 4 weeks before the investigation. Patients under 18 years old, with a previous history of cancer (previous hormone therapy, chemotherapy, radiotherapy), in pregnancy and having lactated within the past 6 months, and with autoimmunological disorders, were excluded from the study. Another exclusion criterion was infections and inflammation diseases diagnosed and antibiotics within 4 weeks before being recruited to the study. Higher concentration of proinflammatory markers such as CRP, Procalcitonin and Il-6, and also CA125, HE4 can be associated with active inflammatory disease. This is the reason for the exclusion of patients with inflammation within 4 weeks.

After confirmation of the presence of a pelvic tumor, in every case, laboratory tests including CA125, HE4, CEA, CA19-9, C-reactive protein, procalcitonin and interleukin-6 and complete blood count were performed. Laboratory tests were performed for differential diagnosis. A 10 mL sample of peripheral blood was obtained from all patients participating in the study. Then, serum samples were separated. Blood samples in a volume of 10 mL after serum isolation were stored at a temperature of 2–8 °C. 

Finally, the histological diagnosis was revealed after the surgical procedure. Suspicion of advanced ovarian cancer was confirmed by biopsy, and then, neoadjuvant chemotherapy introduced or treated first by radical surgery with adjuvant chemotherapy. Laparoscopic surgery was performed in cases of diagnostic procedures or suspected benign tumors.

Analysis was performed after receiving histological results. From further analysis, patients with borderline ovarian tumors and metastatic ovarian tumors were excluded because of selection bias. Higher concentrations of other markers may be observed in the case of different cancers because of metastases located in the ovaries. 

The analyzed population was divided into two groups. The first one consisted of 25 patients with diagnosed ovarian cancer after histological confirmation with a mean age of 61.62 ± 10.27 years (range 32.52–80.82 years). Median age was 62.37 years. Characteristics of clinical stages of ovarian cancer according to the FIGO classification and histological classification are presented in Table 1.

The second group included 54 patients with benign ovarian tumors with a mean age of 52.26 ± 17.99 years (range, 23.97–92.53 years). Median age was 49.32 years. Characteristics of histological types of benign ovarian tumor are presented in Table 2.

Twenty-two patients were excluded from further analysis because of borderline ovarian tumor and ovarian metastases of another cancer. Those patients will be further analyzed in another study. This investigation is concentrated only on the comparison of benign ovarian tumor and ovarian cancer.

No eligible patient declined to participate in this study.

### 2.2. Laboratory Tests

Comparative analysis of complete blood count was based on morphological parameters and complete blood count with leukocyte differentiation, and it was performed using the BC6200 apparatus. C-reactive protein and procalcitonin were assessed by the immunoturbidimetric method with latex agglutination using the ARCHITEKTi 2000SR apparatus.

### 2.3. Immunoenzymatic Analysis

The value of CA125 was assessed from serum using an immunochemical test with chemiluminescent microparticle immunoassay (CMIA) by the Alinity i analyzer. The cut-off value for the serum CA125 level was set at 35 U/mL. The HE4 level for premenopausal women was set at 70 pmol/L and for menopausal women at 140 pmol/L. The ROMA algorithm was further calculated using special methods depending on premenopausal or postmenopausal age based on values of CA125 and HE4 as described by Moore et al. [25].

The cut-off values for serum CEA and Ca19-9 levels were set at 5.0 ng/mL and 37.0 U/mL. Levels were measured with the Alinity i analyzer using immunochemical tests. 

### 2.4. Il-6 Chemiluminescence Analysis

The test for quantitative determination of Il-6 in human serum and plasma is based on a chemiluminescence assay using the MAGLUMI series fully-auto chemiluminescence assay analyzer (including Maglumi 600, Maglumi 800, Maglumi 1000, Maglumi 2000 Plus, Maglumi 4000 Plus, Maglumi X8).

The sample, buffer, ABEI labeled with anti Il-6 monoclonal antibody and magnetic microbeads coated with another Il-6 monoclonal antibody were mixed thoroughly and incubated, forming sandwich complexes. After precipitation in a magnetic field, the supernatant was decanted, then a wash cycle was performed. Subsequently, the Starter 1 + 2 were added to initiate a chemiluminescent reaction. The light signal was measured by a photomultiplier as relative light units (RLUs), which is proportional to the concentration of Il-6 present in the sample (or calibrator/control, if applicable). The cut-off value for serum Il-6 was set at 7 pg/mL.

All normal ranges are presented in Table 3.

### 2.5. Statistical Analysis

Descriptive analysis based on mean, median and standard deviation was performed in MS Excel. The study population was characterized using the Shapiro–Wilk test, non-parametric Mann–Whitney U test and chi-square test. The sensitivity and specificity of markers were calculated using the receiver operating characteristics (ROC) method and the area under the curve (AUC) with 95% confidence intervals (95% CI). Statistical significance of the predictive value of AUC was 0.5. A *p*-value < 0.05 was considered statistically significant in all analyses. Statistical analysis was performed using R programming language version 4.1.2 (R Core Team, Vienna, Austria). For ROC analysis, pROC version 1.18.0 was used. Random forest calculation was done using version 4.7-1.1. For further analysis, “Boot” version 1.3-28 and “Dunn.test” version 1.3.5 were used.

## 3. Results

### 3.1. Role of Markers in Diagnosis of Ovarian Cancer

#### 3.1.1. Sensitivity and Specificity of Tumor Markers in Diagnosis of Ovarian Cancer

In the analyzed population, ovarian cancer was detected in 25 patients with a mean age of 61.62 ± 10.27 years (range, 32.52–80.82 years). Median age was 62.37 years. The control group with a diagnosis of benign ovarian tumors consisted of 54 patients with mean age of 52.26 ± 17.99 years (range, 23.97–92.53 years). Median age was 49.32 years. Marker values were assessed in every case before the final histological result. 

Additionally, maximum concentration of CA125 was 18,345.7 mg/dL, which represents serous ovarian cancer IVA in the FIGO classification, while 606.3 mg/dL was observed in the multilocular benign cyst. The maximum value of HE4 in ovarian cancer was 4725.4 pmol/L for the patients with serous ovarian cancer in IVA, while it was 241.3 pmol/L in patients with teratoma. The ROMA calculation was higher in the ovarian cancer group at 99.78%, while in the control group, it was 85.04%. 

The maximum value of CEA was 263.3 ng/mL, which represented serous ovarian cancer IVA according to the FIGO classification, while it was 4.4 ng/mL for cystadenofibroma. The maximum value of CA19-9 was 635.4 U/mL in endometrioid cancer IIIA according to the FIGO classification. The highest value of CA19-9 was detected in the case of patients with benign ovarian cancer with a histological result of endometriosis and was 4087.1 U/mL. The maximal C-reactive protein concentration was 243 mg/dL for ovarian cancer and 330 mg/dl for benign ovarian tumor with a diagnosis of serous papillary cystadenoma. Procalcitonin was the highest, 40.56 mg/dL, in a patient with serous ovarian cancer IIIC according to the FIGO classification, while it was 0.1 mg/dL in a patient with an ovarian abscess. Otherwise, Il-6 was observed as having a higher value in ovarian cancer, with a maximum of 79.75 ng/mL in a patient with serous ovarian cancer in IIIC, while it was 40.19 ng/mL in the case of a simple ovarian cyst. 

Mean values of the markers CA125, HE4, CEA, CRP, PCT and Il-6 were higher in the group with a diagnosis of ovarian cancer. Only mean concentration of CA19-9 was higher in patients with a benign ovarian tumor. A comparison of concentrations of markers in ovarian cancer and benign ovarian tumors is presented in Table 4.

For better estimation in diagnosis of ovarian cancer, correlations of Il-6 concentration with other parameters were performed. It revealed that Il-6 is a more predictable marker of diagnosis of ovarian cancer with age, CA125, HE4, ROMA, WBC, hemoglobin, number of platelets, C-reactive protein, procalcitonin and NLR (neutrophil-to-lymphocyte ratio) and PLR (platelet-to-lymphocyte ratio). All correlations are presented in Table 5.

To sum up, the most significant factors in ovarian cancer are the ROMA algorithm, Ca125, HE4 and RDW-CV as blood count parameters. Number of platelets, MCH as median concentration, Il-6 and MCHC are less important in detection of ovarian cancer, which was statistically significant in Spearman correlation.

Additionally, sensitivity and specificity of every marker were calculated in every case in the diagnosis of ovarian cancer using ROC analysis with AUC calculations, which are presented in Table 6.

**Figure 1:**
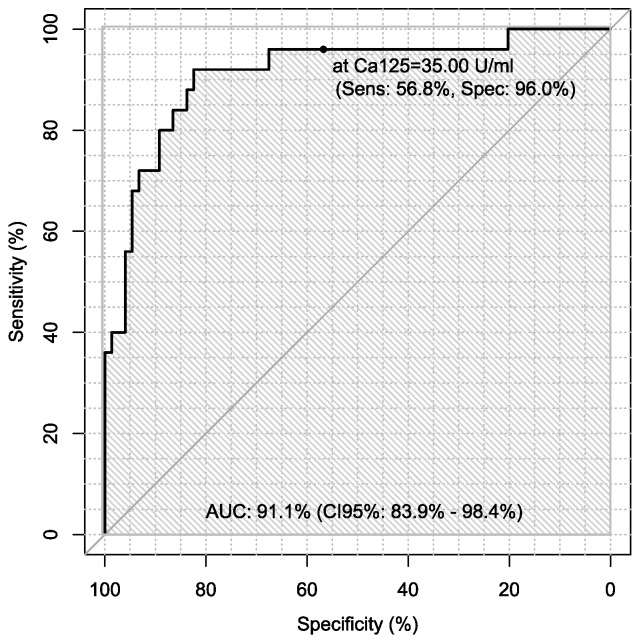
ROC-AUC of CA125.

**Figure 2:**
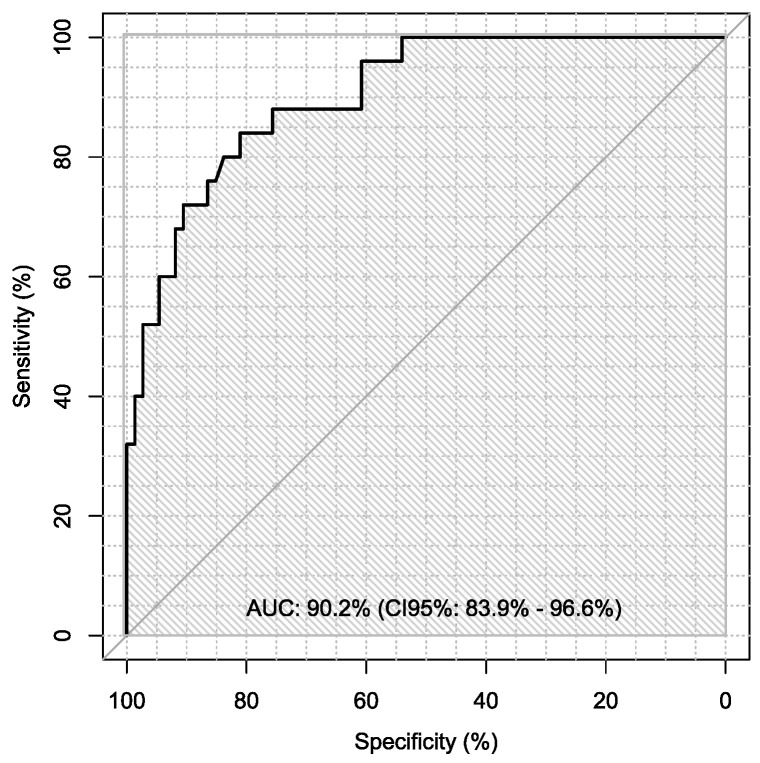
ROC-AUC of HE4.

**Figure 3:**
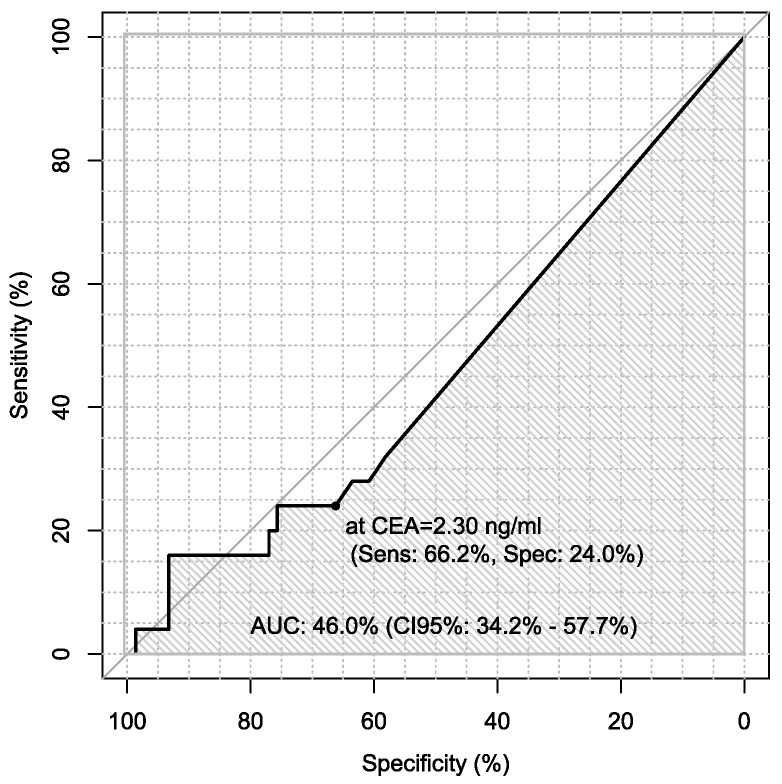
ROC – AUC of CEA.

**Figure 4:**
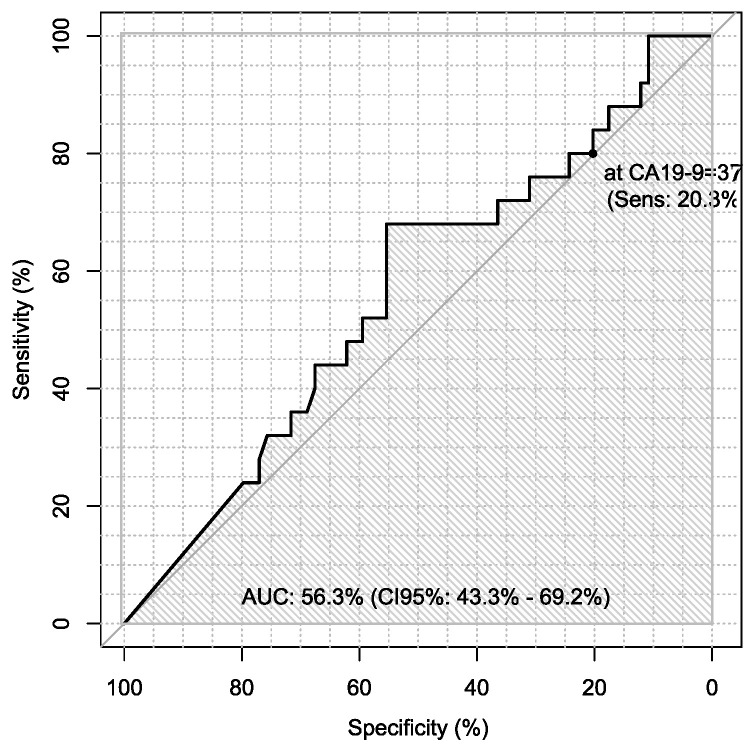
ROC – AUC of CA19-9.

**Figure 5:**
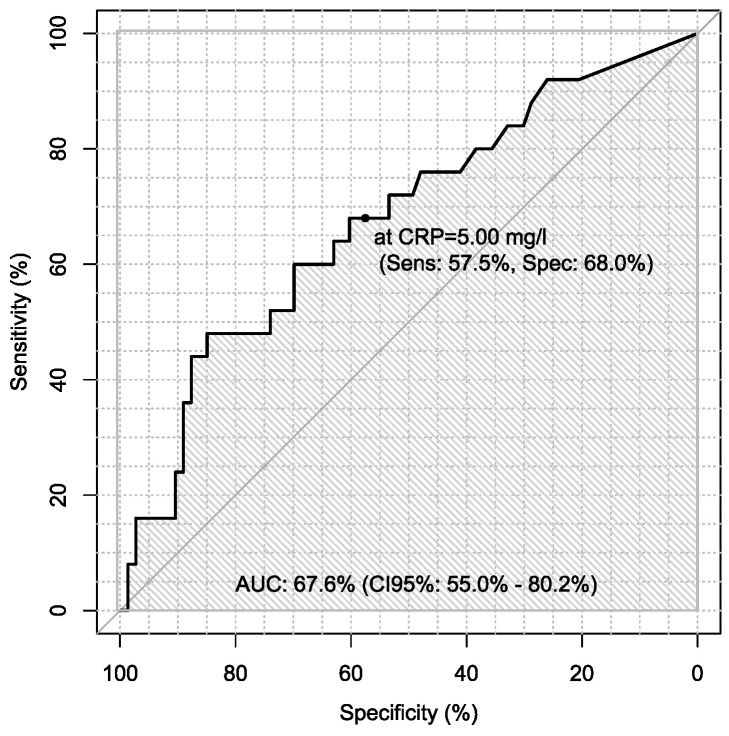
ROC – AUC of CRP.

**Figure 6:**
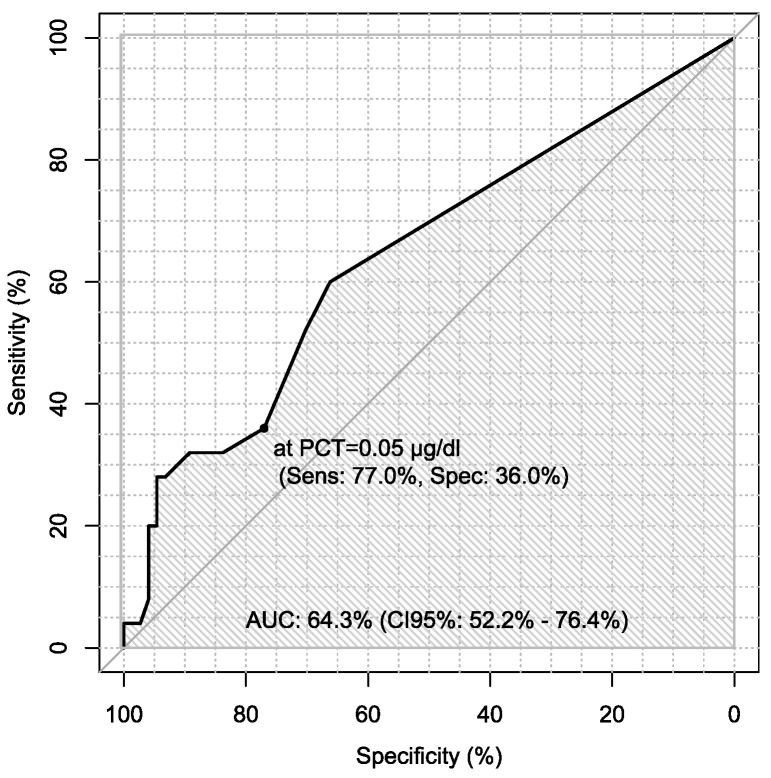
ROC – AUC of Procalcitonin.

**Figure 7:**
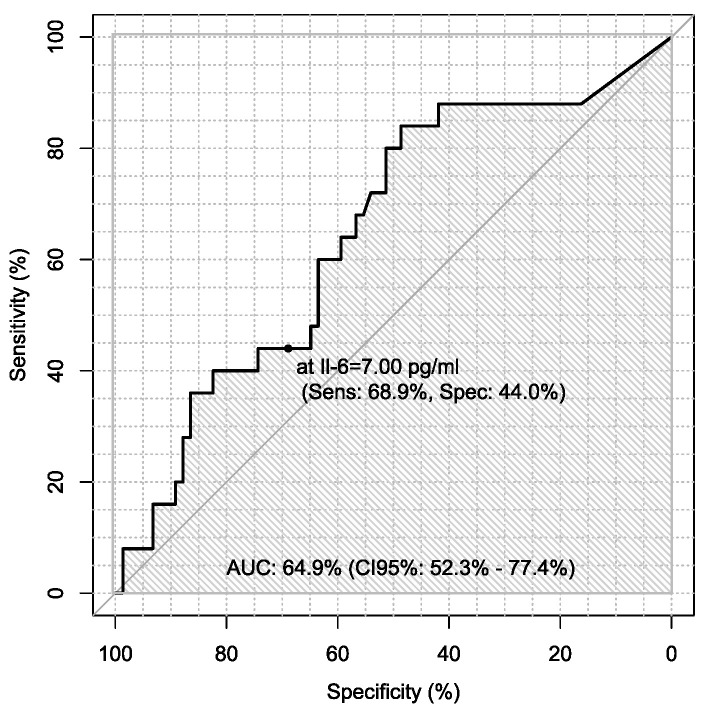
ROC – AUC of Il-6.

Sensitivity and specificity of markers are illustrated in Figure 1, Figure 2, Figure 3, Figure 4, Figure 5, Figure 6 and Figure 7.

#### 3.1.2. Histological Type of Ovarian Cancer

In our investigation of a population of patients with ovarian cancer, three different histological types were detected. The most frequent was serous (14 patients, 56%); the rest were endometrioid (9 patients, 36%) and clear-cell carcinoma (2 patients, 8%).

Only Il-6 is statistically relevant to the histological serous type of ovarian cancer, which was revealed using Spearman correlation. Data are presented in Table 7.

**Figure 8:**
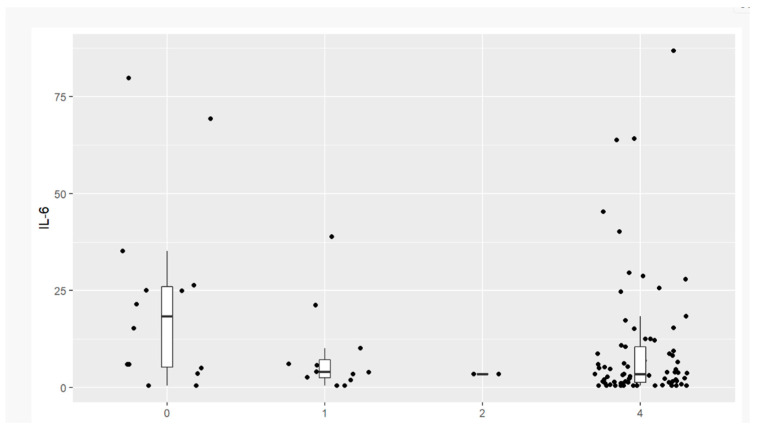
Correlation of Il-6 and histological type of ovarian cancer: (0) serous type, (1) endometroid, (2) clear-cell carcinoma, (4) benign pelvic tumors, non-malignant ones.

Statistical significance was observed according to histological type and the value of Il-6. In most cases, patients with serous ovarian cancer had elevated values of Il-6. The correlation is visualized in Figure 8.

According to histological grading, to differentiate malignancy of cancer using G (grade), 8% (2 patients) were G1, 28% (7 patients) were G2, and 64% (16 patients) were G3. Histological grading was statistically significant for CA125, HE4, CEA, CRP, PCT and Il-6. Data are presented in Table 7.

#### 3.1.3. FIGO Classification

After surgery, in every case of ovarian cancer, clinical staging according to the FIGO classification was confirmed. For further analysis, patients were divided into two groups: early stage of ovarian cancer (stage I and II according to the FIGO classification) and late stage (III and IV, FIGO). In our investigation, 4 patients (16%) were in the early stage of ovarian cancer and 21 patients (84%) in the late stage. All data for population characteristics are presented in Table 1.

**Figure 9:**
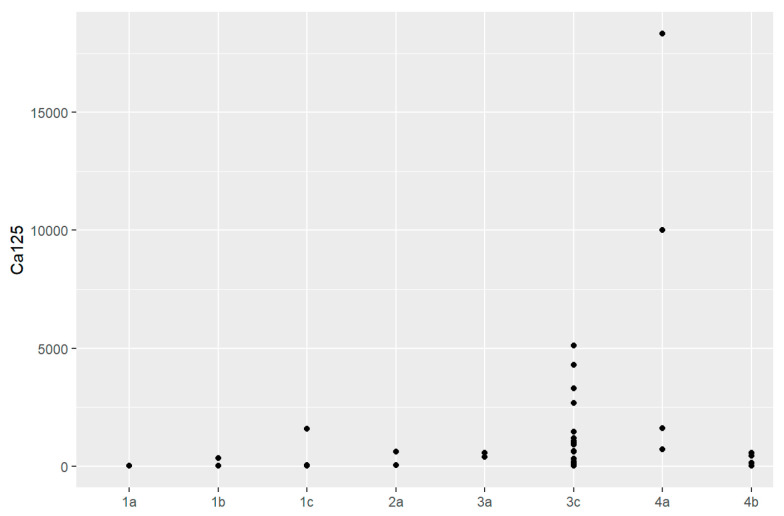
Correlation of CA125 and FIGO classification of ovarian cancer (Spearman, *p*-value < 0.001%).

**Figure 10:**
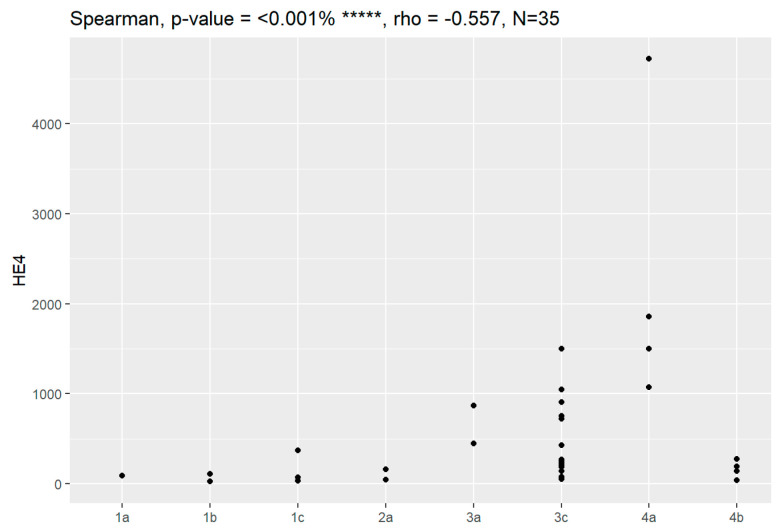
Correlation of HE4 and FIGO classification of ovarian cancer. ***** Statistically significant.

**Figure 11:**
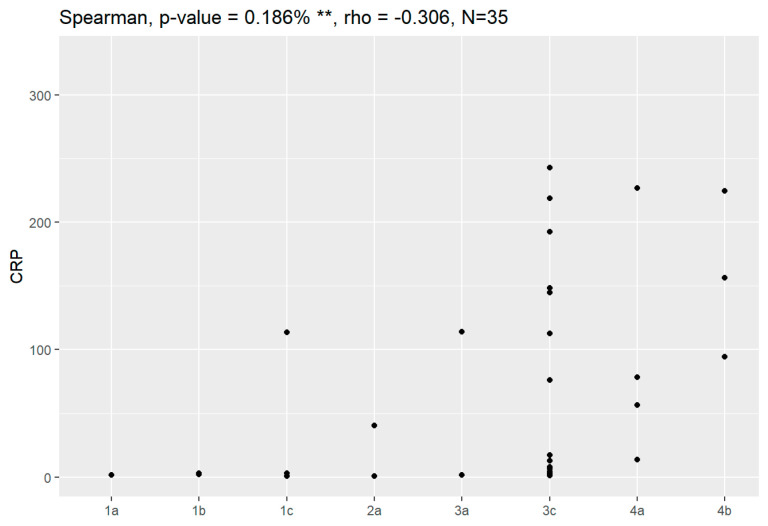
Correlation of CRP and FIGO classification of ovarian cancer. ** Statistically significant.

**Figure 12:**
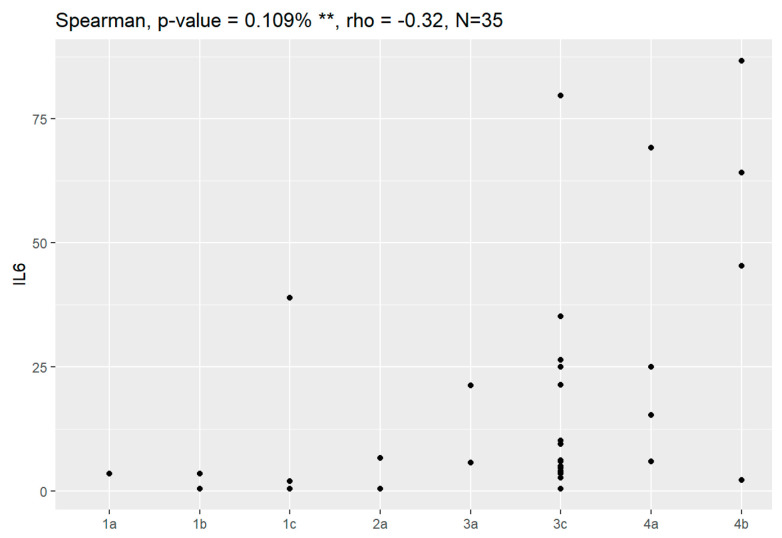
Correlation of Il-6 and FIGO classification of ovarian cancer. ** Statistically significant.

**Figure 13:**
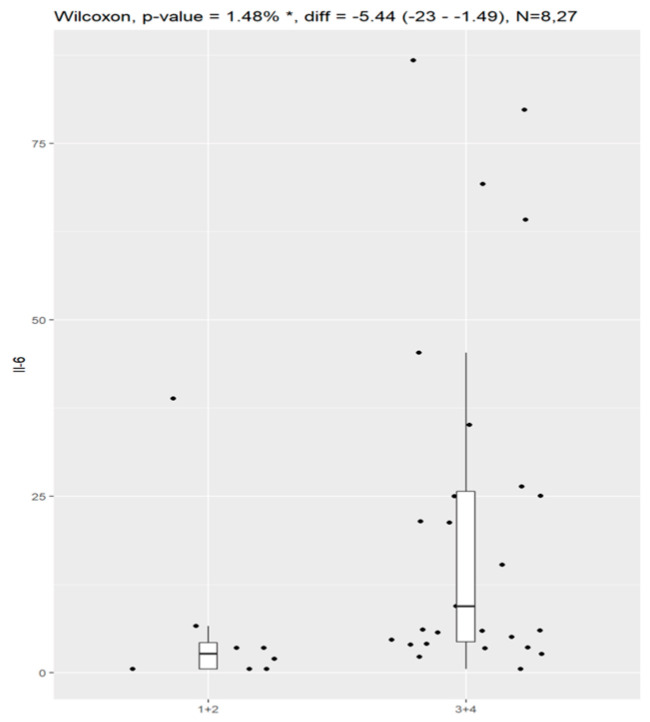
Correlation of Il-6 and FIGO classification of ovarian cancer: (1 + 2)—early stages of ovarian cancer, stages I and II; (3 + 4)—advanced stages of ovarian cancer, stages III and IV. * Statistically significant.

In comparison between early (I and II according to FIGO classification) and late (III and IV according to FIGO classification) stages of ovarian cancer, statistically significant impacts were detected for the algorithm Ca125, HE4, CRP and Il-6. Values of these markers were higher for an advanced stage of the disease. Correlations of FIGO classification and markers are presented in Figure 9, Figure 10, Figure 11, Figure 12 and Figure 13.

#### 3.1.4. BRCA Mutation

Ovarian cancer was confirmed histologically in 25 patients from the analyzed population. After surgery, in every case of ovarian cancer, genetic tests of the ovarian tumor were performed. The presence of a BRCA1 mutation was detected in 6 cases (24% of all groups with ovarian cancer). No homologous recombination deficiency was confirmed. The remaining patients of this group do not have a genetic background of ovarian cancer. No mutation was confirmed.

The mean age of patients with a BRCA mutation was 56.8 ± 8.37 years (range, 44.79–64.81 years) vs. 63.14 ± 10.53 years (range, 32.52–80.82 years) in the group with no BRCA mutation. Median age was 58.54 years vs. 63.84 years. Higher mean concentrations of the markers CA125, HE4, CEA, Ca19-9, CRP, PCT were observed in patients with no BRCA mutation. ROMA calculations were also higher in the group with no BRCA mutation. Only the mean value of Il-6 was observed as higher in BRCA mutation patients; but there was no statistically significant association of higher concentration of Il-6 with the presence of a BRCA mutation. In the group with the presence of a BRCA mutation, one patient was observed with a normal value of Il-6 (3.58 ng/mL). In two patients with a diagnosis of endometrioid ovarian cancer, concentrations of Il-6 were 38.87 and 10.1 ng/mL (stage IC and IIIC, respectively). In 4 other patients with BRCA, mutation values were 3.58, 24.98, 15.3 and 21.43 ng/mL (clinical stage of ovarian cancer IIIC, IIIC, IVA, IIIC, respectively). There was no statistically significant association between the histological type of ovarian cancer and the presence of BRCA mutation. Four patients with ovarian cancer and BRCA mutation were classified as stage IIIC according to FIGO. Characteristics and correlations of both groups according to genetic background are presented in Table 8.

### 3.2. Impact of Concentration of Il-6 on Survival of Patients

Every patient with a diagnosis of ovarian cancer was treated with chemotherapy based on carboplatin and paclitaxel. In 9 cases, neoadjuvant chemotherapy was introduced. After that, in 5 cases, radical surgery was performed. Four patients were not operated on because of progression of ovarian cancer during neoadjuvant chemotherapy. The rest of the patients with ovarian cancer underwent radical hysterectomy with bilateral salpingoovariectomy, bilateral lymphadenectomy and omentectomy with further chemotherapy based on carboplatin and paclitaxel. Apart from patients with a progression of the disease, in 7 cases, recurrence of ovarian cancer was detected and the second line of chemotherapy was applied.

#### 3.2.1. Recurrence of Ovarian Cancer

**Figure 14:**
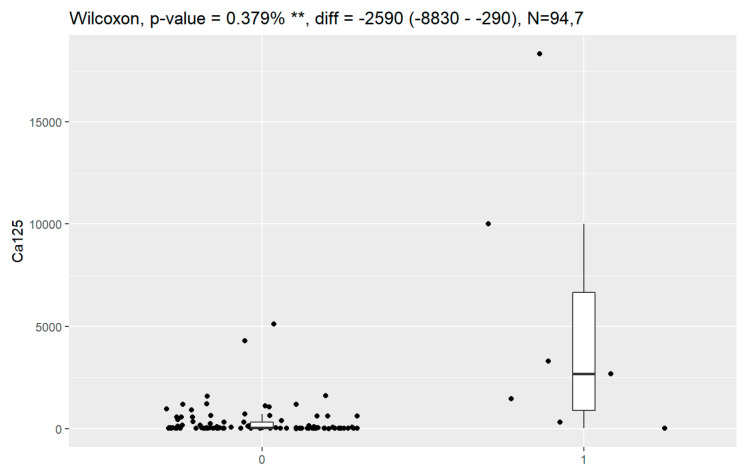
Correlation of CA125 and HE4 and recurrence of ovarian cancer (0—no recurrence, 1—recurrence). ** Statistically significant.

**Figure 15:**
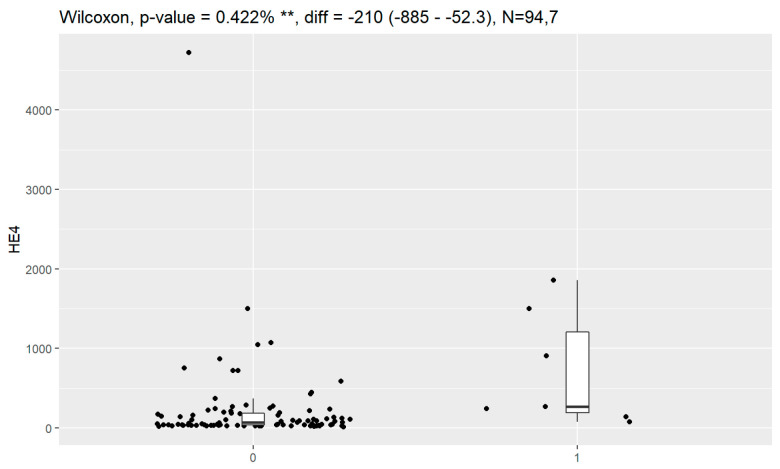
Correlation of CA125 and HE4 and recurrence of ovarian cancer (0—no recurrence, 1—recurrence). ** Statistically significant.

Recurrence was observed in 7 patients (28%) from the group with ovarian cancer. Six patients were diagnosed with serous ovarian carcinoma (4 with stage IIIC and 2 with stage IVA) and one patient with clear-cell ovarian carcinoma in stage IIIC according to the FIGO classification. Two of the patients with a recurrence of ovarian cancer were BRCA mutation positive. Patients with recurrence were older (higher mean age of 64.63 vs. 60.45 years in the other patients). Patients with recurrence had a higher value of CA125 at the time of diagnosis, with a mean value of 4347.62 (range: 13.2–18,345.7). Mean values of the markers CA125 and CEA were higher at diagnosis of ovarian cancer. Otherwise, values of HE4, Ca19-9, CRP, PCT and Il-6 were lower than in the group without recurrence. Higher levels of CA125 and HE4 at the time of diagnosis of ovarian cancers are statistically significant for future recurrence of the disease. Data of correlations between tumor markers before diagnosis and recurrence of ovarian cancer are presented in Figure 14 and Figure 15.

#### 3.2.2. Progression of Ovarian Cancer

In 6 cases from the analyzed population with ovarian cancer, we observed platin-resistant ones with progression during chemotherapy based on carboplatin and paclitaxel (4 patients after neoadjuvant chemotherapy and 2 after radical surgery with adjuvant chemotherapy). This was the reason for modification of the treatment. In the progression group, there were 5 patients with serous ovarian cancer (3 with IIIC FIGO and 2 with IVA FIGO) with a mean age of 56.81 ± 15.035 years (range: 32.52–74.78 years) vs. 63.14 ± 8.23 years (range: 44.79–80.82 years). What is interesting, in comparison to the rest of the group, patients with progression had higher values of HE4, CRP, PCT and Il-6, which was statistically significant with the markers HE4, CRP and Il-6. Progression of ovarian cancer was also statistically significantly associated with levels of CA125 and CEA at the time of diagnosis of this disease. Comparison of concentrations of markers in the case of the progression of ovarian cancer is presented in Table 9.

#### 3.2.3. Overall Survival

During the investigation, there were 8 deaths due to ovarian cancer: 7 patients with serous ovarian cancer (2 with IVA FIGO, 5 with stage IIIC FIGO) and 1 patient with clear-cell ovarian cancer in stage IIIC, according to the FIGO classification, with a mean age of 59.08 ± 13.54 (range: 32.52–80.82). This group consisted of 5 patients with progression of ovarian cancer from the onset with a mean age of 53.21 years (range: 32.52–64.81 years) and mean overall survival of 207 days (range: 31–617 days) and 3 patients with recurrence with a mean age of 68.85 years (range: 61.62–73.85 years) and a mean overall survival of 546 days (range: 336–712 days). In the group with a progression of the disease who died, there were 2 patients with a BRCA mutation at age 49.75 and 64.81 years, respectively, with an overall survival of 617 days and 268 days, respectively.

Patients who died during the investigation were characterized by higher levels of HE4, CRP, PCT and IL-6 at the time of diagnosis of ovarian cancer compared to the others, which is presented in Table 10.

**Figure 16:**
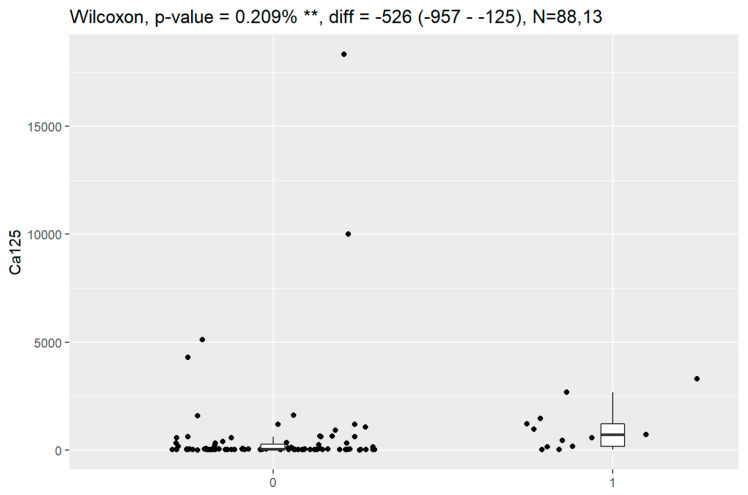
Level of CA125 and correlation with death (0—no death, 1—death). ** Statistically significant.

**Figure 17:**
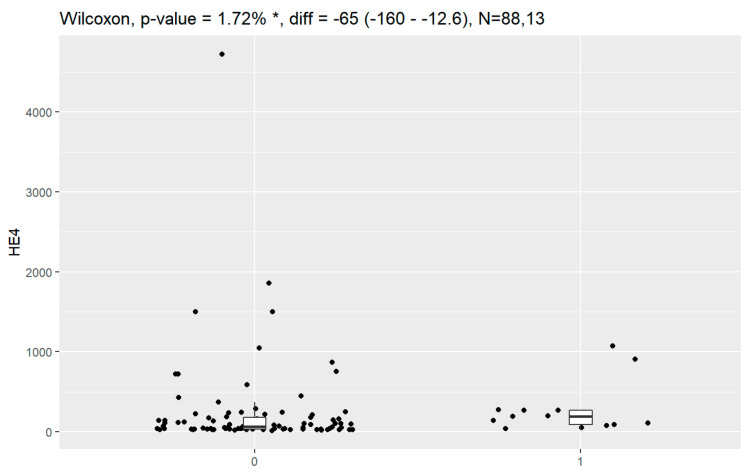
Level of HE4 and correlation with death (0—no death, 1—death). * Statistically significant.

**Figure 18:**
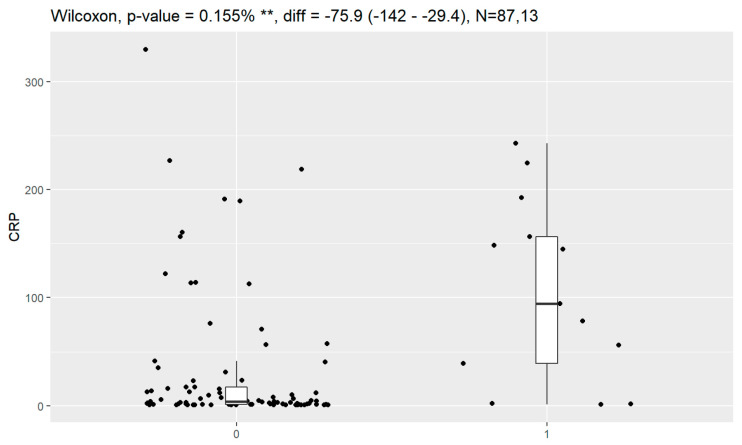
Level of CRP and correlation with death (0—no death, 1—death). ** Statistically significant.

**Figure 19:**
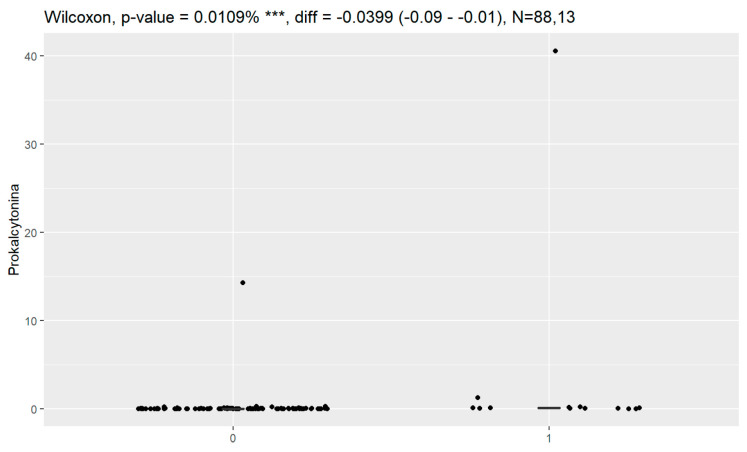
Level of procalcitonin and correlation with death (0—no death, 1—death). *** Statistically significant.

**Figure 20:**
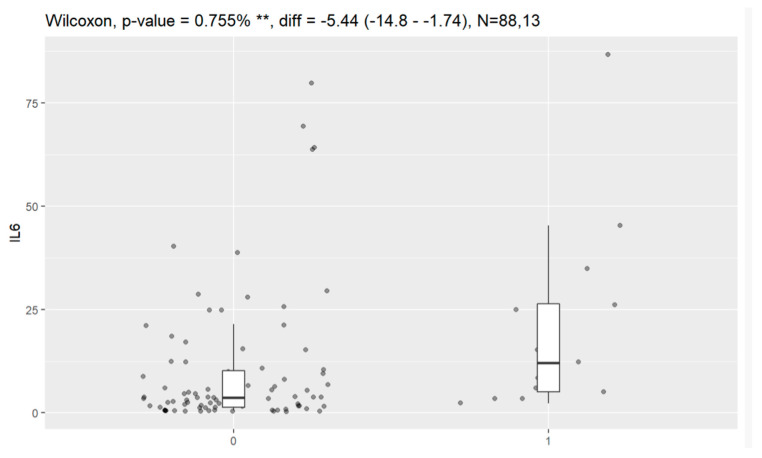
Level of Il-6 and correlation with death (0—no death, 1—death). ** Statistically significant.

Statistically significant associations of levels of tumor markers at the time of diagnosis and death were observed for CA125, HE4, CRP, PCT and Il-6. Data are presented in Figure 16, Figure 17, Figure 18, Figure 19 and Figure 20.

Overall survival (OS) and progression-free survival (PFS) were calculated in every case. Longer overall survival was correlated with lower values of Il-6. In the case of higher concentration of Il-6, OS and PFS were shorter. Every patient who died because of the progression of ovarian cancer demonstrated a higher value of Il-6.

Statistically significant associations of overall survival were observed with levels of CA125, HE4, CRP, PCT and Il-6 measured at the time of diagnosis of ovarian cancer.

**Figure 21:**
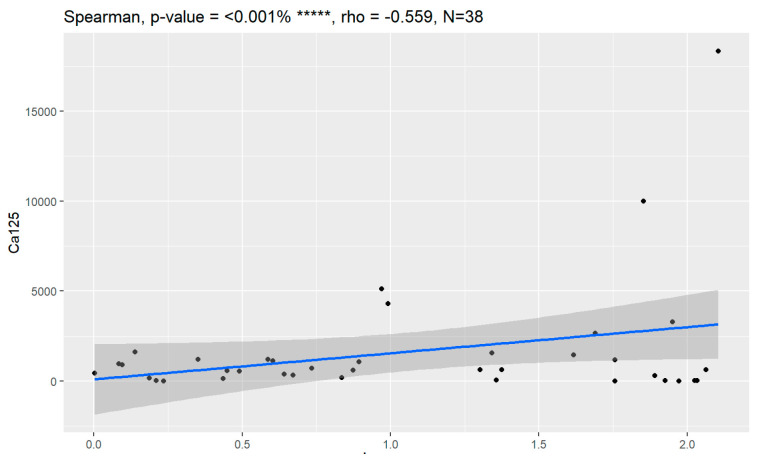
Overall survival and level of CA125. ***** Statistically significant.

**Figure 22:**
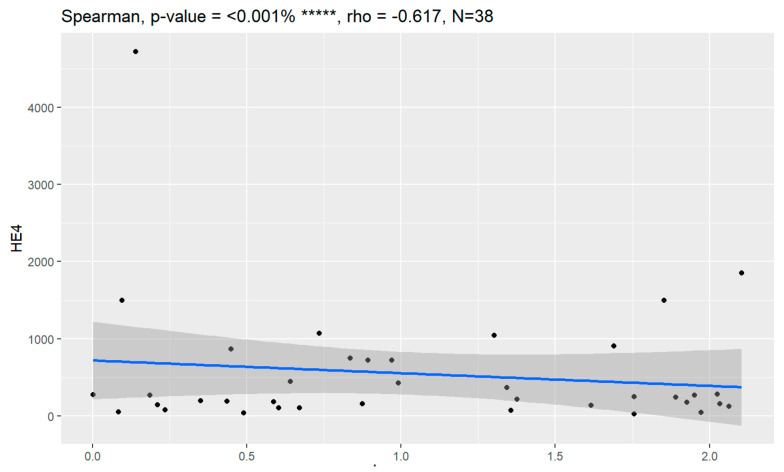
Overall survival and level of HE4. ***** Statistically significant.

**Figure 23:**
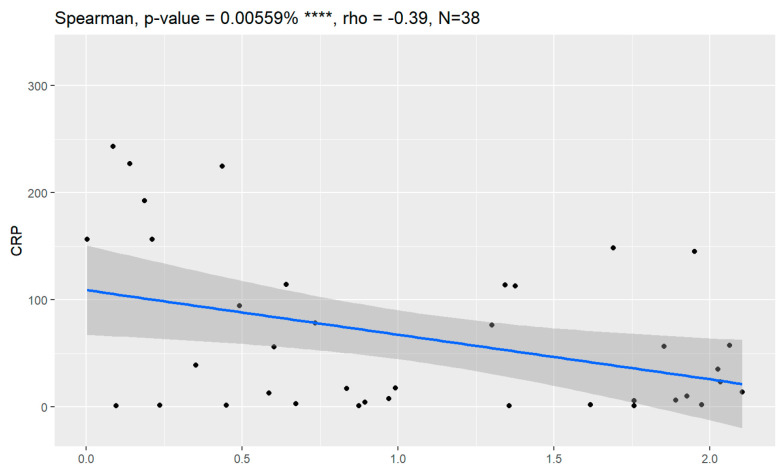
Overall survival and level of CRP. **** Statistically significant.

**Figure 24:**
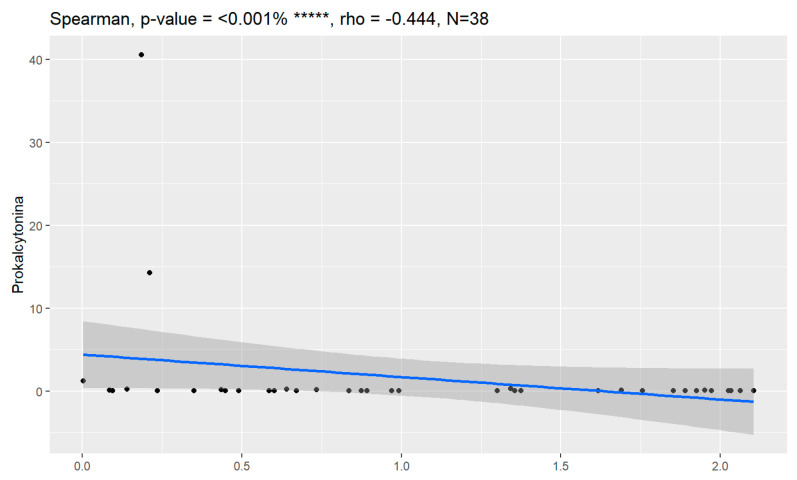
Overall survival and procalcitonin. ***** Statistically significant.

**Figure 25:**
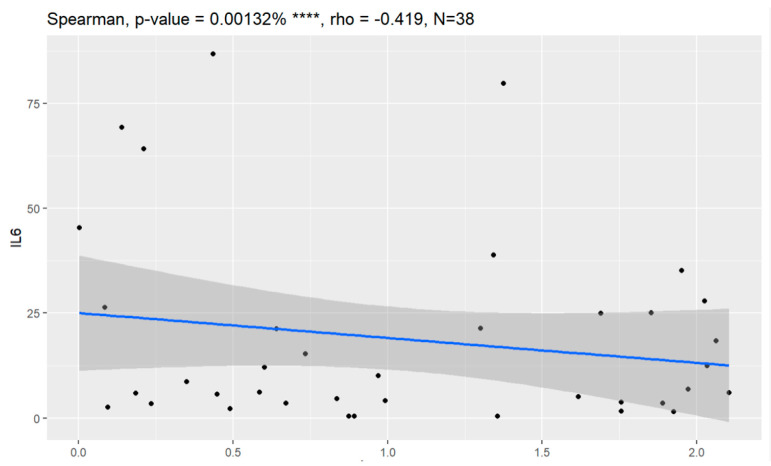
Overall survival and Il-6. **** Statistically significant.

**Figure 26:**
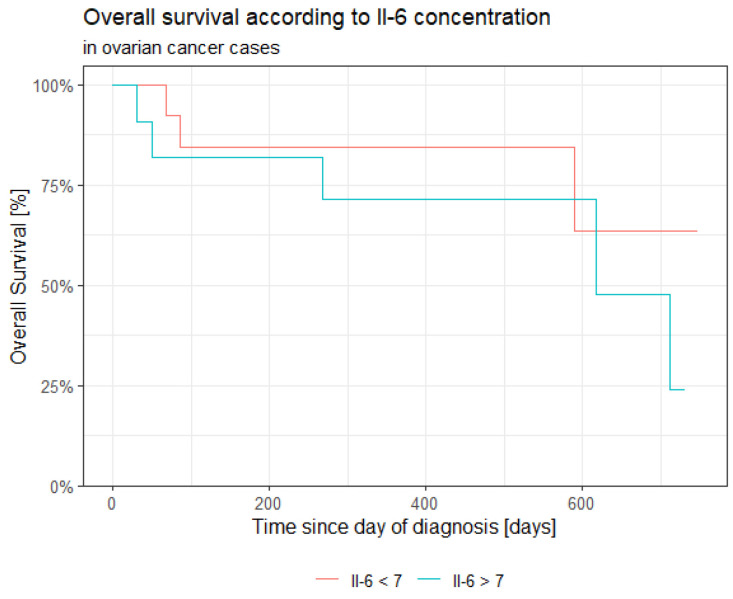
Overall survival and Il-6 (Kaplan–Meier estimate).

Overall survival of group of patients with ovarian cancer is presented in Figure 21, Figure 22, Figure 23, Figure 24, Figure 25 and Figure 26.

## 4. Discussion

Pelvic tumors can be a symptom of different diseases. New markers are sought for detection of ovarian cancer in the early stage, which allows for longer overall survival and longer progression-free survival. Lack of symptoms and deficiency of specific markers are reasons for diagnosis in advanced stages of ovarian cancer, which gives a poor prognosis.

The most common, specific and sensitive markers of diagnosis of ovarian cancer are CA125 and HE4, which are calculated together using the ROMA algorithm [25]. The ROMA algorithm and risk of malignancy index (RMI) were described by Anton et al. in the classification of ovarian masses. Unfortunately, RMI has a lower predictive value, so this method is not used as a specific one [26]. Additionally, the markers CEA and CA19-9 are determined to differentiate digestive system tumors. Lertkhachonsuk et al. in 2020 observed a positive correlation between elevated serum CA125, followed by Ca19-9 and CEA in mucinous ovarian tumors [27]. In our study, we observed only 2 patients with benign mucinous cystadenoma with higher levels of Ca19-9 of 4087.1 and 1141.8 U/mL. However, we confirmed a statistically significant association of level of CEA and diagnosis of endometriosis (*p*-value =0.024). Nevertheless, there is no evidence of a high predictive value of CEA and Ca19-9 in ovarian cancer.

The tumor microenvironment is focused on the inflammatory process. Prognostic potential is sought in inflammatory markers. One such indicator is Il-6, which occurs as a pleiotropic marker with dual function of the immune system as pro- and anti-inflammatory effects. By STAT3 activation, Il-6 introduces expression of genes conducive to tumor progression. This is a phenomenon of breast, colorectal and head and neck cancer [28,29,30]. Fu et al. also observed activation of STAT3 by Il-6 in gastric cancer progression and a higher level of this cytokine in this kind of patient [31]. In our study, patients with progression of ovarian cancer were detected with higher levels of Il-6, CRP and PCT, which confirmed the inflammatory background of neoplastic disease.

Shi et al. observed correlations with peripheral blood-based markers and survival in advanced non-small cell lung cancer patients who received immunotherapy-based treatments and revealed an increase of Il-6 during treatment and an association with poor prognosis [32]. In our investigation, a higher level of Il-6 was also correlated with poor prognosis and statistically significantly associated with death, overall survival and progression-free-survival.

What is more, Cristiani et al. observed the level of Il-6 in patients with melanoma and confirmed that serum levels of Il-6 identify progression of this disease. On the other hand, they reported that a lower concentration of Il-6 was associated with longer PFS and OS [33]. In our study, we observed similar associations.

Dobrzycka et al. reported that Il-6 and C-reactive protein (CRP) were poor markers for prognosis of overall survival and disease-free survival (DFS). In their study, median values of Il-6 and CRP were higher than in the control group and were 11.5 pg/mL (range: 3.4–62.6) vs. 2.9 mg/L (range: 1.1–12.3) and 9.51 pg/mL (range: 0.3–129.2) vs. 1.2 mg/L (range: 0.1–11.5). In our study, median values of Il-6 and CRP were, respectively, 16.76 pg/mL (range: 0.5–79.75) and 75.02 mg/L (range: 1–243) for patients with ovarian cancer and 5.92 pg/mL (range: 0.5–40.19) vs. 26 mg/L (range: 1–330) for women with a benign pelvic tumor. For better estimation of the inflammatory influence, we decided to also evaluate procalcitonin. The median value of procalcitonin in patients with ovarian cancer was 2.21 ng/mL (range: 0.02–40.56) vs. 0.04 ng/mL (range: 0.02–0.1). According to Dobrzycka et al., a higher level of inflammatory parameters correlated with reduced survival and disease-free survival [34]. We also observed reduced survival in the case of increasing values of these factors. On the other hand, Poole et al. observed that a higher level of C-reactive protein indicated the role of inflammation in pathophysiology of ovarian cancer [35]. Yoshida et al. confirmed CRP as an inductor of immunosuppressive melanoma [36]. Recent studies showed correlations between renal cell, lung, breast cancer, pancreatic cancer and poor prognosis and overall survival [37,38,39,40]. In our study, death, progression and overall survival were also correlated statistically significantly with higher level of CRP.

On the other hand, there is only one study using procalcitonin as a marker of ovarian cancer. Coccolini et al. recorded serum and peritoneal marker concentrations during hyperthermic intraperitoneal chemotherapy. In their study, the most significant variations were in procalcitonin and Il-6 levels [41]. In our study, we used procalcitonin as a marker of differentiation of ovarian cancer. Sensitivity and specificity of PCT, Il-6 and CRP were, respectively, 36% and 77%; 46.8% and 77.8%; 68% and 57.5%. Our study showed that sensitivity of PCT was the lowest, so it seems not to be a good marker in diagnosis of ovarian cancer.

Han et al. described the correlation of Il-6 and HE4 as a new diagnostic panel [42]. In our study, we also noted the statistical significance of these two markers but counted separately. On the other hand, after creating special qualifications, we discovered a statistically significant correlation of Il-6 concentration with ROMA, CA125, HE4, RDW, WBC and lymphocytes using analysis with “Boot” version 1.3-28 and “Dunn.test” version 1.35. There was no influence of procalcitonin on the sensitivity of Il-6 in the diagnosis of ovarian cancer.

In 2021, Pawlik et al. performed a study comparing the clinical importance of Il-6, Il-8 and TNF-α in differentiation of ovarian carcinoma and benign ovarian tumor. They detected the statistically significant role of Il-8 and TNF-α and higher levels in malignant tumors. They did not find a role of Il-6 in the differentiation of tumors [43]. In our study, we observed statistical significance of Il-6 in the diagnosis of ovarian cancer (*p*-value = 0.0412).

In 2020, de Lima et al. noted higher levels of cytokines such as Il-2, 5, 6, 8 TNF alpha and Il-10 in malignant ovarian tumors in peritoneal fluid than in benign ones [44]. Unfortunately, we did not have the possibility to assess Il-6 in peritoneal fluid so we concentrated only on serum samples.

Micheli et al. compared levels of Il-6 before and 30 days after surgery, while we assessed only levels before surgery. Cut-off values for Il-6 for malignant tumors were 11.3 pg/mL and 7.2 pg/mL for the control group, while in our study, the values were 16.76 pg/mL and 5.9 pg/mL [45]. Adjuvant treatment reduced Il-6 but did not influence the value of other markers. In our study, we did not assess values of Il-6 after surgery, but we noted a higher level of Il-6 in advanced stages which progressed during adjuvant chemotherapy.

Concentration of Il-6 in peritoneal fluid is higher than in serum, but it seemed not to be possible to carry out staging according to the FIGO classification based only on IL-6 [46]. On the other hand, in our study, we observed a higher concentration of serum Il-6 in advanced stages of ovarian cancer and it was correlated with poor prognosis of the disease. Kampan et al. in their study observed 33 patients with ovarian cancer and compared them with patients with benign ovarian tumors and healthy ones. In our investigation, ovarian cancer was diagnosed in 25 cases, and our results were similar to Kampan’s study. The concentration of Il-6 was higher in advanced stages of ovarian cancer, so it may be a clinically reliable marker for differentiation of high-grade ovarian cancer and patients with normal ovaries. What is more, Kampan et al. revealed predictive power in combination of Il-6 with RMI score, ROMA, HE4 and CA125. However, they consider Il-6 as a useful diagnostic tool in the diagnosis of ovarian cancer [47].

Furthermore, Liu et al. revealed Il-6 as a prognostic marker in postoperative follow-up. The level of Il-6 was higher in post-operative measurements compared to VEGF [17]. What is more, Seki et al. suggested in their study that ovarian clear cell carcinoma tumors with high Il-6 levels are candidates for bevacizumab therapy. The mechanism is based on the support of Il-6 on the anti-angiogenic activity of bevacizumab by suppressing Ang1 expression and promoting dependence on VEGF for angiogenesis [48]. In our study, there was no detection of Il-6 after surgery. However, we observed progression during adjuvant treatment in cases with a higher concentration of Il-6.

Amer et al. performed a systematic review and meta-analysis of the impact of a higher level of Il-6 (23.88 pg/mL) in late stages of ovarian cancer compared to earlier ones (16.67 pg/mL). In our study, the value of Il-6 was also higher in the population of more advanced OC [49]. In our study, in the comparison between early (I and II according to the FIGO classification) and late stages of ovarian cancer (III and IV according to the FIGO classification), there was a statistically significant impact of the algorithm HE4, Ca125, CRP, PCT and Il-6. Amer et al. did not confirm a correlation between level of Il-6 in serum and peritoneal fluid. However, the mean level of Il-6 of advanced stages of ovarian cancer was also higher than in earlier ones. What is more, values of cytokines were higher in peritoneal fluid than in serum [49].

Zhang et al. explored the synergistic effect of hypoxia and higher concentration of Il-6, which promote invasion of cancer cells. This mechanism can be also associated with higher concentration of Il-6, which we have observed in patients in an advanced stage of ovarian cancer [50].

On the other hand, Xu et al. observed that serum CA19-9 and HE4 are valuable in the diagnosis of endometriosis-associated ovarian cancer in the case of a large ovarian tumor and age over 42 years [51]. In our study, we did not find a significant correlation between the histological type of ovarian cancer and HE4 and Ca19-9. Possibly it is because of the larger amount of serous ovarian cancer in the analyzed population.

Zeng et al. performed a meta-analysis of prospective studies and provided evidence that elevated levels of CRP are associated with an increased risk of ovarian cancer, while levels of circulating Il-6, TNFα and soluble TNFR2 are not [52]. In our study, we confirmed a statistically significant association between the diagnosis of ovarian cancer and levels of CA125, HE4, CRP, PCT and Il-6.

Additionally, there was no study of a correlation between concentration of Il-6 and the presence of BRCA mutation. In our investigation, the results are not statistically significant, which may be associated with a few patients being BRCA positive. Further investigations should be done in this area.

Ovarian cancer with metastases in the abdominal cavity has a poor prognosis. Inflammatory markers are indicators of inflammation but can also be an indicator of distant metastases. Warli et al. detected that higher level of Il-6 correlated with metastases in lymph nodes in bladder cancer [53]. On the other hand, Hashemzehi et al. observed an association of Il-6 -174G>C polymorphism with the risk of ovarian and also cervical cancer [54]. In 2020, Ahmed et al. published a study about the association of TGF-ß1 rs 1880242 and Il-6 SNPs in the inflammatory process in ovarian cancer recurrence. Heterozygosity of TGF-ß1 rs 1880242 indicates a decreasing risk of ovarian cancer [55].

In our study, we also observed and evaluated the concentration of Il-6 in advanced stages of ovarian cancer with metastases in the abdominal cavity and lungs and in metastatic tumors located in the ovaries. We are planning to describe the correlation which we noted between these groups of patients in a further publication.

Additionally, in 2021, Yan et al. described a novel potential targeted therapy for epithelial ovarian cancer based on the dual blockade of EGFR and Il-6 STAT3 signaling by miR-146b. STAT3 activation has recently been suggested to be correlated with resistance of cells to anti-EGFR therapy [56]. Because of the activation of STAT3 by Il-6, it seems that the blockade of Il-6 and inhibition of this marker prolong survival of patients with ovarian cancer. On the other hand, Pettersen et al. revealed that activin A in an autocrine and paracrine manner impact on secretion of Il-6 from the cancer cells. A higher concentration of Il-6 stimulated by activin A can cause transformation of cancer cells to distant metastases [57].

## 5. Conclusions

Il-6 can be a prognostic marker of advanced stages of ovarian cancer and neoplastic diseases. Patients with ovarian cancer and a higher value of Il-6 had shorter overall survival and shorter progression-free survival or even progression of disease after diagnosis.

Further investigations are necessary in diagnostics of pelvic tumors and to shed further light on the role of Il-6.

## Figures and Tables

**Table 1:** Characteristics of population with ovarian cancer.

Characteristic		Number of Patients
Clinical stages of ovarian cancer		
Early (I and II)		
	IA	0
	IB	1
	IC	2
	IIA	1
	IIB	0
Advanced (III and IV)		
	IIIA	2
	IIIB	0
	IIIC	15
	IVA	4
	IVB	0
Histological stages of ovarian cancer		
	G1	2
	G2	7
	G3	16
Histological types of ovarian cancer		
	Serous	14
	Endometrioid	9
	Clear cell	2

**Table 2:** Characteristics of population with benign pelvic tumors.

Histological Type	Number of Patients
Serous cystadenoma	21
Mucinous cystadenoma	2
Fibroma	8
Endometrial cyst	11
Dermoid cyst	6
Hemorrhage cyst	2
Ovarian abscess	1
Hydrosalpinx	1
Fibroids/myomas	3

**Table 3:** Cut-off values of markers.

Tumor Marker	Cut-Off Value
CA125	35 U/mL
HE4	Premenopausal 70 pmol/L
	Postmenopausal 140 pmol/L
CEA	5.0 ng/mL
CA19-9	37.0 U/mL
C-reactive protein	5.0 mg/L
Procalcitonin	0.02 ng/mL
Il-6	7.00 pg/mL

**Table 4:** Concentrations of markers in ovarian cancer and benign ovarian tumors.

	Markers	Ovarian Cancer	Benign Tumors
CA125	Range; Median	13.2–18,345.7; 716.9	5.4–606.3; 26
	Mean	2000.91 ± 3813.37	64.43 ± 116.58
HE4	Range; Median	53.1–4725.4; 319.65	15.29–241.3
	Mean	694.03 ± 982.97	58.48 ± 49.01
ROMA	Range; Median	15.28–99.9; 96.91	0.9–85.04; 5.32
	Mean	86.92 ± 21.00	17.37 ± 22.65
CEA	Range; Median	1.8–263.3; 4.6	1.7–4.4; 2.7
	Mean	36.93 ± 91.5	2.85 ± 0.84
CA19-9	Range; Median	2.4–635.4; 6.8	2.3–4087.1; 10.65
	Mean	68.67 ± 158.24	142.67 ± 647.58
CRP	Range; Median	1–243; 37.05	1–330; 3.65
	Mean	75.02 ± 82.93	26 ± 64.84
PCT	Range; Median	0.02–40.56; 0.04	0.02–0.1; 0.04
	Mean	2.21 ± 9.28	0.043 ± 0.027
Il-6	Range; Median	0.5–79.75; 5.99	0.5–40.19; 2.33
	Mean	16.76 ± 20.73	5.92 ± 8.74

**Table 5:** Correlation of Il-6 concentration in patients with diagnosis of ovarian cancer.

Variable	*p* Value	rho
Age at the time of the study	0.00799	0.382
Diagnosis of ovarian cancer	4.12	0.204
Ca125	<0.001	0.46
HE4	<0.001	0.588
ROMA	<0.001	0.548
CEA	0.173	0.308
Ca 19-9	77	0.0295
WBC	<0.001	0.482
Lymphocytes	0.276	−0.295
Hgb	0.00966	−0.378
HCT	0.013	−0.372
MCV	6.83	−0.182
PLT	0.0279	0.354
MPV	94.8	−0.00663
NLR	<0.001	0.485
PLR	<0.001	0.446
CRP	<0.001	0.723
PCT	<0.001	0.67
Overall Survival	0.00132	−0.419

**Table 6:** Sensitivity and specificity of markers in diagnosis of ovarian cancer.

Marker	Sensitivity	Specificity	AUC
Ca125	76.6 (63.8–87.2)	63 (50–75.9)	0.817 (0.731–0.904)
Ca19-9	29.8 (17–42.6)	87 (77.8–94.4)	0.561 (0.446–0.676)
CEA	40.4 (27.7–55.3)	75.9 (64.8–87)	0.609 (0.508–0.709)
Il-6	46.8 (31.9–61.7)	77.8 (66.7–87)	0.731 (0.631–0.830)
C-reactive protein	68.0 (48.0–84.0)	57.5 (46.6–68.5)	0.676 (0.550–0.802)
Procalcitonin	36.0 (16.0–56.0)	77.0 (67.6–86.5)	0.643 (0.522–0.764)

**Table 7:** Correlation of tumor markers and characteristics of ovarian cancer.

	CA125	HE4	CEA	CA19-9	CRP	PCT	Il-6
	*p*-Value	*p*-Value	*p*-Value	*p*-Value	*p*-Value	*p*-Value	*p*-Value
Diagnosis of OC	<0.001	<0.001	0.586	0.386	0.0123	0.018	0.0412
FIGO classification	<0.001	<0.001	0.0931	0.532	0.00186	0.009	0.00109
Correlation with endometriosis	0.458	0.000534	0.0244	0.441	0.179	0.017	0.00495
Histologic classification (G)	<0.001	<0.001	0.04	0.787	0.00176	0.0184	0.00754
Histological type	0.138	0.25	0.574	0.447	0.181	0.557	0.0973
BRCA mutation	0.514	0.0923	0.579	0.37	0.504	0.263	0.239

**Table 8:** Characteristics of population and correlations with markers according to genetic background.

		BRCA Mutation (+)	BRCA Mutation (−)
Age	Range; Median	44.79–64.81; 58.54	32.52–80.82; 63.84
	Mean	56.8 ± 8.37	63.14 ± 10.53
Ca125	Range; Median	306.2–5120.9; 1146	13.2–18,345.7; 620.1
	Mean	1837.52 ± 1821.36	2058.69 ± 4352.56
HE4	Range; Median	239.1–1069.4; 815.05	53.1–4725.4; 249.5
	Mean	725.38 ± 351.16	683.58 ± 1127.18
ROMA	Range; Median	85.77–99.34; 98.06	15.28–99.9; 92.59
	Mean	96.21 ± 5.19	84 ± 23.3
CEA	Range; Median	1.8–2.8; 2.3	2.1–263.3; 6.85
	Mean	2.3 ± 0.71	48.48 ± 105.27
Ca19-9	Range; Median	6.7–126.8; 7.2	2.4–635.4; 6.65
	Mean	46.9 ± 69.2	72.76 ± 171.17
CRP	Range; Median	6.5–148.4; 77.25	1–243; 15.65
	Mean	71.85 ± 56.6	76.08 ± 91.42
PCT	Range; Median	0.02–0.28; 0.075	0.02–40.56; 0.04
	Mean	0.11 ± 0.1	3.19 ± 11.22
Il-6	Range; Median	3.58–38.87; 18.36	0.5–79.75; 5.69
	Mean	19.04 ± 12.38	16.04 ± 22.98

**Table 9:** Concentrations of tumor markers and survival of patients.

		Recurrence	Non-Recurrence	Death	Alive	Progression	Non-Progression	Population
Age	Range	49.75–73.85	32.52–80.82	32.52–73.85	44.79–80.82	32.52–74.78	44.79–80.82	32.52–80.82
	Mean	64.63	60.45	59.08	62.81	56.81	63.14	61.62
	Median	64.36	62.3	63.22	62.37	58.81	62.37	62.37
CA125	Range	13.2–18,345.7	49.3–5120.9	13.2–3293.8	49.3–18,345.7	180.2–2670	13.2–18,345.7	13.2–18,345.7
	Mean	4347.62	1172.77	1364.71	2340.34	1091.55	2321.97	2000.99
	Median	2063.4	636	1212.9	620.1	842.95	636	716.9
HE4	Range	74.9–1854.3	53.1–4725.4	53.1–4725.4	69.7–1854.3	53.1–4725.4	69.7–1854.3	53.1–4725.4
	Mean	579.98	732.05	938.05	572.03	1245.08	510.35	694.03
	Median	252.15	397.1	267.6	397.1	676.3	252.15	319.65
ROMA	Range	15.28–99.9	30.52–99.78	15.28–99.78	30.52–99.9	74.54–99.78	15.28–99.9	15.28–99.9
	Mean	83.65	88.2	82.16	89.17	91.46	85.49	86.92
	Median	97.45	95.09	93.26	96.91	95.72	96.91	96.91
CEA	Range	2.8–263.3	1.8–8.8	2.8–7.5	1.8–263.3	2.8–7.5	1.8–263.3	1.8–263.3
	Mean	89.7	5.28	5.15	47.53	5.15	47.53	36.94
	Median	3	6.2	5.15	4.6	5.15	4.6	4.6
CA19-9	Range	6.5–126.8	2.4–635.4	2.5–126.8	2.4–635.4	2.4–126.8	4.1–635.4	2.4–635.4
	Mean	33.67	84.83	23.4	95.08	27.8	83.27	68.674
	Median	10.35	5.4	6.5	12.3	3.3	10.55	6.8
CRP	Range	1.4–148.4	1–243	1.4–243	1–218.9	78.2–243	1–218.9	1–243
	Mean	53.41	83.92	129.72	47.67	167.23	44.28	75.02
	Median	13.8	76.3	146.7	13.4	170.55	10.5	37.05
PCT	Range	0.02–0.11	0.02–40.56	0.02–40.56	0.02–0.28	0.11–40.56	0.02–0.28	0.02–40.56
	Mean	0.05	3.48	5.17	0.07	6.91	0.06	2.21
	Median	0.03	0.08	0.11	0.04	0.21	0.04	0.04
Il-6	Range	3.45–35.13	0.5–79.75	3.45–69.25	0.5–79.75	5.94–69.25	0.5–79.75	0.5–79.75
	Mean	14.75	17.54	23.18	13.74	27.19	13.47	16.76
	Median	5.99	6.03	20.14	5.69	23.13	5.04	5.99

**Table 10:** Correlations of ovarian tumor markers and patients’ survival.

	CA125	HE4	CEA	Ca19-9	CRP	PCT	Il-6
	*p*-Value	*p*-Value	*p*-Value	*p*-Value	*p*-Value	*p*-Value	*p*-Value
Recurrence	0.00379	0.00422	0.733	0.619	0.276	0.412	0.119
PFS	<0.001	<0.001	0.0475	0.993	0.00213	0.0602	0.0186
Overall survival	<0.001	<0.001	0.0673	0.976	0.000559	<0.001	0.000132
Death	0.00209	0.0172	0.377	0.0109	-	0.0109	0.00755

## Data Availability

The data presented in this study are available on request from the corresponding author. The data are not publicly available due to ethical restrictions.
